# Use of Daridorexant among Patients with Chronic Insomnia: A Retrospective Observational Analysis

**DOI:** 10.3390/jcm12093240

**Published:** 2023-05-01

**Authors:** Scott G. Williams, Domingo Rodriguez-Cué

**Affiliations:** 1 Center for Military Psychiatry and Neuroscience, Walter Reed Army Institute of Research, Silver Spring, MD 20910, USA; 2 Departments of Medicine and Psychiatry, Uniformed Services University of the Health Sciences, Bethesda, MD 20814, USA; 3 SleepCues PA, 5603 Duraleigh Rd, Suite 105, Raleigh, NC 27612, USA

**Keywords:** insomnia, insomnia medications, DORA, daridorexant, switching

## Abstract

Insomnia is the most prevalent sleep disorder, affecting millions worldwide and taking a heavy toll on patient health with significant social and economic impact. Even though there are multiple different types of insomnia medications and behavioral therapies, there are still many individuals for whom treatment remains ineffective. The objective of this retrospective study was to analyze the effectiveness of daridorexant in a cohort of chronic insomnia patients largely transitioned from GABA-A positive allosteric modulators (benzodiazepines, zolpidem or eszopiclone) or other frequently prescribed insomnia medications (including trazodone, atypical antipsychotics or tricyclic antidepressants). A total of 86 patients were treated in the course of ordinary practice and the primary analytic endpoint was the change in Insomnia Severity Index (ISI) score following ≥ 30 nights of treatment with daridorexant. Results from 80 of the 86 patients with full data (65% female, mean age 53.5 years, 18.8% with comorbid obstructive sleep apnea, 91.3% transitioned from a different medication) showed a mean improvement in ISI score of 7.0 ± 0.54 points (SEM) (*p* < 0.0001) from 18.0 to 11.0. Overall, 78% of the cohort demonstrated a clinically meaningful improvement as defined by at least a six-point drop in ISI. Total sleep time increased by 54 ± 1.0 min (SEM) (*p* < 0.0001) from 6.0 h to 6.9 h. Mean sleep latency decreased by 23.9 ± 2.4 min (SEM) (*p* < 0.0001) from 58.8 min to 34.9 min. Wake after sleep onset decreased by 31.6 ± 3.2 min (SEM) (*p* < 0.001) from 42.8 min to 11.3 min. Sleep efficiency improved by 10.5 ± 1.1% (SEM) (*p* < 0.0001) from 79.3% to 89.8%. No significant adverse events were noted during the study duration. Keeping in mind this study’s limitations, these data suggest that for insomnia patients with an incomplete response to current therapy, switching to daridorexant is safe and may be an effective alternative treatment.

## 1. Introduction

Insomnia manifests acutely as a result of long-distance travel, a traumatic event, or an illness, or it can be considered chronic when it has persisted for at least 3 months at a frequency of at least 3 times per week [1]. Insomnia disorder presents with a range of symptoms including difficulty falling asleep, difficulty staying asleep, poor sleep quality, and early morning awakening leading to daytime impairment [1,2]. In addition to those symptoms, the complications associated with issues such as obstructive sleep apnea (OSA) or other comorbid conditions, adverse drug effects, and daytime somnolence should be considered; in this way, one begins to understand why there is no “one-size-fits-all” treatment. This makes treatment of insomnia a highly complex issue. Cognitive Behavioral Treatment for Insomnia (CBT-I) is the first-line treatment for all patients who meet criteria for chronic insomnia. Though it is highly effective, many patients are prescribed pharmacologic therapy.

In 2020, 8.4% of adults took sleep medication in the last 30 days to help them fall or stay asleep, according to a January 2023 data brief published by the Centers for Disease Control and Prevention [3]. Clinicians, therefore, must be familiar with the various therapeutic targets of the different insomnia medications. Given that gamma-aminobutyric acid (GABA) is the most prominent inhibitory neurotransmitter [4], many of the older sedative hypnotics act on this receptor to produce somnolence. Benzodiazepines (e.g., triazolam, flurazepam, estazolam, quazepam, and temazepam) are positive allosteric modulators (PAMs) at the GABA-A receptor, thus enhancing its activity [5]. When safety concerns arose regarding the potential for abuse or addiction with benzodiazepine treatment [6,7,8,9,10,11] and the increased risk of falls and/or cognitive impairment in elderly insomnia patients [12,13,14,15], prescription of non-benzodiazepine (non-BZD) GABA-A receptor PAMs otherwise known as “Z drugs” (zolpidem, zaleplon, and eszopiclone) gained in popularity. However, in 2016, the U.S. Food and Drug Administration (FDA) issued a black-box warning on the potential increased risk of sleepwalking, sleep driving, and complex sleep behaviors after the use of Z-drugs [16].

In addition, a number of medications are prescribed off-label for the treatment of insomnia. Despite lacking an FDA indication for insomnia, the sedating antidepressant trazodone is one of the most commonly prescribed medications for chronic insomnia. Other commonly used off-label medications include mirtazapine, quetiapine, and amitriptyline. These medications are regularly prescribed when mood disorders are simultaneously present [17,18]. Many providers and patients, however, are not aware that these medications do not have an FDA indication for the treatment of insomnia.

Dual orexin receptor antagonists (DORAs) are the newest class of FDA-approved insomnia medications. DORAs have a different mechanism of action compared with previously approved sedative hypnotics. Instead of enhancing inhibitory neurotransmitter activity, DORAs antagonize wake-promoting neural pathways. They exert their effect by blocking the binding of orexin-A and orexin-B neuropeptides to the orexin receptor, thus decreasing arousal and wakefulness [19]. Since orexin-producing neurons are located exclusively in the hypothalamus, DORAs have a more narrowly targeted mechanism of action compared to other drug classes [20,21].

Suvorexant was the first FDA-approved DORA [22] and has been shown to improve all sleep parameters [23]. Like suvorexant, lemborexant reduces sleep onset latency, increases total sleep time, and improves sleep efficiency [24]. Daridorexant, the newest DORA with a shorter terminal half-life than its predecessors (8 h vs. 12 h for suvorexant and 17–19 h for lemborexant), was approved in January 2022 [25,26]. Initial clinical study results have demonstrated a favorable side effect profile with improvement in sleep parameters over existing insomnia medications [27,28,29]. However, there is only a small amount of data available regarding its relative efficacy to older sedative hypnotics.

In this retrospective analysis, we report results from a case series that includes both newly diagnosed insomnia patients and previously diagnosed insomnia patients switching from GABA-A PAMs, trazodone, atypical antipsychotics or tricyclic antidepressants to daridorexant.

## 2. Materials and Methods

The primary study endpoint of this analysis was the change in insomnia symptom severity as measured by the Insomnia Severity Index (ISI) prior to switching and after at least 30 nights of daridorexant use. The ISI is a 7-item self-report questionnaire assessing the nature, severity, and impact of insomnia [30,31,32]. The usual recall period is the “last month” and the dimensions evaluated are:

1. Severity of sleep onset;
2. Sleep maintenance;
3. Early morning awakening problems;
4. Sleep dissatisfaction;
5. Interference of sleep difficulties with daytime functioning;
6. Noticeability of sleep problems by others;
7. Distress caused by difficulty sleeping.

A 5-point Likert scale is used to rate each item (e.g., 0 = no problem; 4 = very severe problem). The 7-item questionnaire is scored between 0 and 28 [31]. ISI scores are interpreted as follows: Absence of insomnia (0–7); Sub-threshold insomnia (8–14); Moderate insomnia (15–21); Severe insomnia (22–28).

A 6-point reduction in ISI can be regarded as representing a clinically meaningful improvement in individuals with insomnia [33].

Secondary endpoints were derived from patient self-reported sleep data that were received as part of the clinicians’ usual medical practice and often included time to sleep initiation (sleep onset latency (SOL)), total time awake after initially falling asleep (wake after sleep onset (WASO)), and total sleep time (TST). The percentage of time asleep in bed divided by the total time in bed (sleep efficiency (SE)) was calculated from the raw data. Clinically meaningful differences for subjective sleep parameters were determined according to the AASM Clinical Practice Guidelines; a mean change ≥ 30 min for TST, a mean change ≥ 20 min for SOL, a mean change ≥ 30 min for WASO and a mean change ≥ 10% for SE [2].

### 2.1. Patient Population

**Figure 1:**
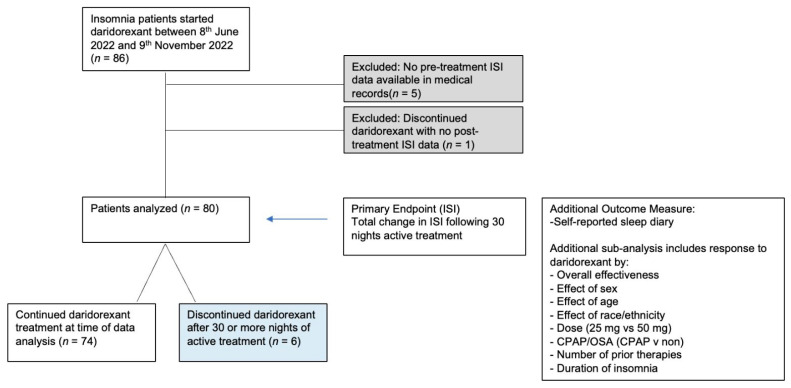
Flow chart of patient population. ISI = insomnia severity index; CPAP = continuous positive airway pressure; OSA = obstructive sleep apnea.

The analysis, which was conducted in December 2022, reviewed medical records from two separate multidisciplinary sleep disorder clinics and included all patients who were initiated on daridorexant from 8 June 2022 to 9 November 2022, (*n* = 86). All patients were offered behavioral sleep medicine treatment as standard care following initial diagnosis of insomnia. There was no ISI data on 5 patients recorded in their medical records prior to starting daridorexant. There was 1 patient who discontinued daridorexant within 30 days of initiating treatment without evidence of any post-treatment ISI data. Thus, the data set of patients with baseline ISI data and after at least 30 days of treatment with daridorexant is *n* = 80 (Figure 1). Among that group, 74 patients continued daridorexant through the data analysis time period, while 6 patients discontinued the medicine (after at least 30 days of treatment and with post-treatment data in their medical records). These six patients were still included in the overall data analysis (*n* = 80) to prevent any selection bias.

In total, 63 patients were switched from another pharmacotherapy for insomnia, typically because they had incompletely responded to the other therapies. Six patients were naïve to insomnia medications. Eleven patients did not have a clear record of prior pharmacotherapy in the medical record. All patients were prescribed this insomnia pharmacotherapy as a routine part of their treatment; prescribing physicians were unaware that patient data would be used as part of a retrospective analysis. All data were anonymized prior to analysis.

### 2.2. Sample Characteristics

Demographics collected from both sleep medicine clinic cohorts included the following: age, sex, race, daridorexant dosage (50 mg or 25 mg at bedtime), whether they were using continuous positive airway pressure (CPAP) for obstructive sleep apnea, the duration of their insomnia, and the number and type of previous medications (if any) they had used prior to switching to daridorexant.

The following self-reported measurements via a sleep diary were also typically collected both prior to switching and at least 30 nights post-switch:

1. Subjective measures of daytime function, including fatigue, sleepiness, concentration, mood, and anxiety.
2. Subjective measures of sleep, including total sleep time (TST), sleep onset latency (SOL), wake after sleep onset (WASO) and sleep efficiency (SE).

### 2.3. Statistical Analysis

All statistical analyses were carried out using GraphPad Prism 9.5.0 (730), Boston, USA. Specific analysis methods appropriate to each data set are noted in the figure legends. The patients’ characteristics are reported as percentages or summarized and tabulated.

## 3. Results

In total, 52 females and 28 males ranging from 24 to 82 years of age were analyzed. Beyond the data analysis time period, 74 patients continued on daridorexant treatment, and 6 received the medicine for at least 30 days but eventually discontinued daridorexant (Table 1).

### 3.1. Insomnia Severity Index (ISI) Data

#### 3.1.1. ISI Scores before and after 30 Days or More of Daridorexant Treatment

**Figure 2:**
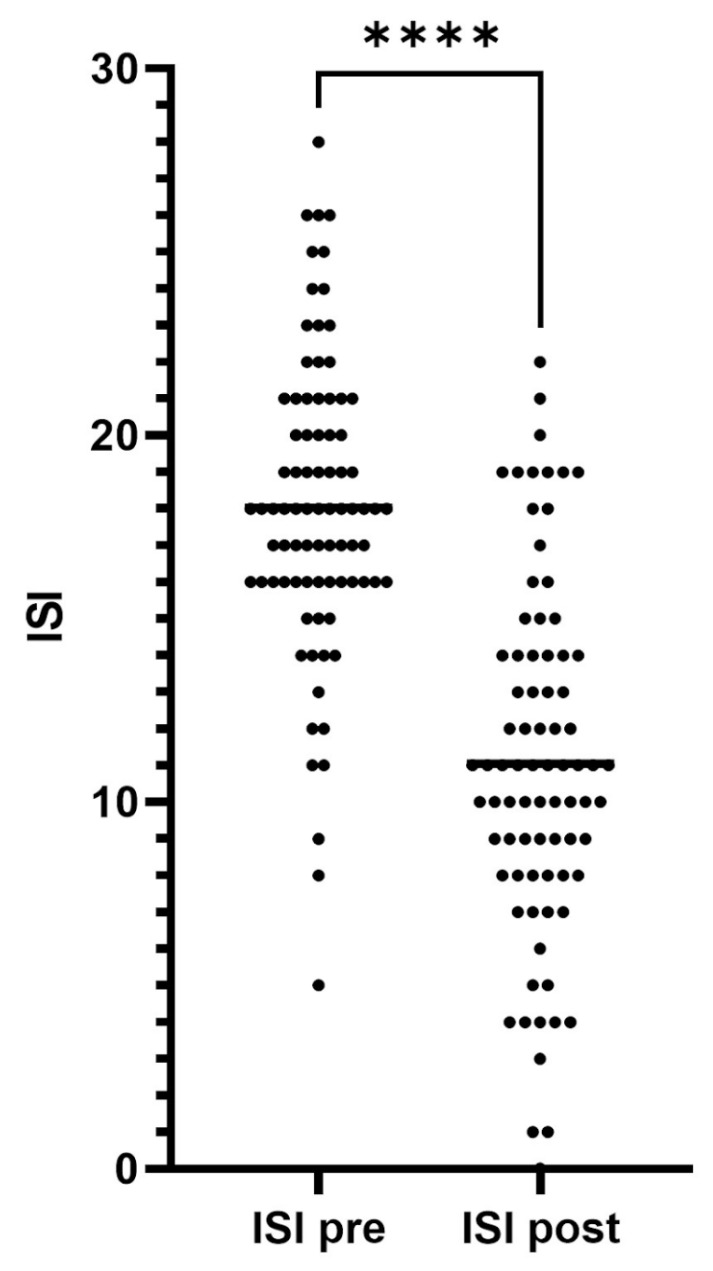
ISI scores, before and after 30 days or more of daridorexant treatment (*n* = 80). Mean ISI after 30 days of 11.0 is significantly lower than pre-daridorexant mean ISI score of 18.0 **** denotes *p* < 0.0001 as analyzed using Wilcoxon matched pairs signed rank statistical test.

Of the patients whose data was analyzed (*n* = 80), 68 patients were treated with a 50 mg dose and 12 patients were treated with a 25 mg dose. Immediately prior to starting or switching to daridorexant, the overall mean ISI score was 18.0 with a standard deviation of 4.1. Following at least 30 consecutive nights of treatment (ranging from one to four months), the mean ISI score was 11.0 with a standard deviation of 4.9 (0.53 SEM). This represents a statistically significant, clinically meaningful improvement in median ISI score of 7.0 ± 0.54 (SEM) (*p* < 0.0001) (Figure 2). While the initial intent of the study was to examine the 1 month change in ISI, there were 3-month follow-up data available for 59 of the 80 patients. Within this subset, the ISI showed no statistical change when comparing the 1-month ISI (11.56 ± 4.23) with the 3-month ISI (11.07 ± 3.95), *p* < 0.53. Overall, 51 of 59 patients (86.4%) remained on daridorexant at 3-month follow-up.

#### 3.1.2. Effect of Sex on ISI

**Figure 3:**
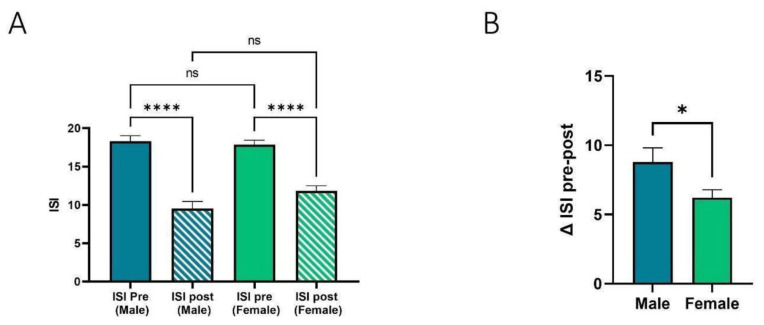
The impact of sex on daridorexant effectiveness. (**A**) Mean ISI score, before and after 30 days or more of daridorexant treatment separated by sex. Both male (*n* = 28) and female (*n* = 52) patients experienced a significant improvement in ISI over the treatment period, as determined using Kruskal–Wallis multiple comparative analysis. **** denotes *p* < 0.0001. (**B**) Mean difference in ISI score after treatment, separated by sex (*n* = 80). Male patients experienced a greater improvement in ISI than females, with mean change in ISI scores of 8.8 (male) vs. 6.2 (female). * denotes *p* < 0.05, as determined using Kruskal–Wallis multiple comparative analysis. ns = non-significant.

The impact of sex on daridorexant effectiveness was examined (Figure 3). The analysis group consisted of 28 males and 52 females. Mean pre-treatment ISI score was 18.3 for males and 18.1 for females. Mean post-treatment ISI scores were 9.5 and 11.9, respectively. This represents a change in mean ISI of 8.8 in the male population (*p* < 0.0001), and a change in mean ISI of 6.2 in the female population (*p* < 0.0001). Males therefore experienced a greater reduction in ISI than females (*p* < 0.05). It is worth noting that 32% of males reported CPAP use compared to only 11.5% of females.

#### 3.1.3. Effect of Age on ISI

**Figure 4:**
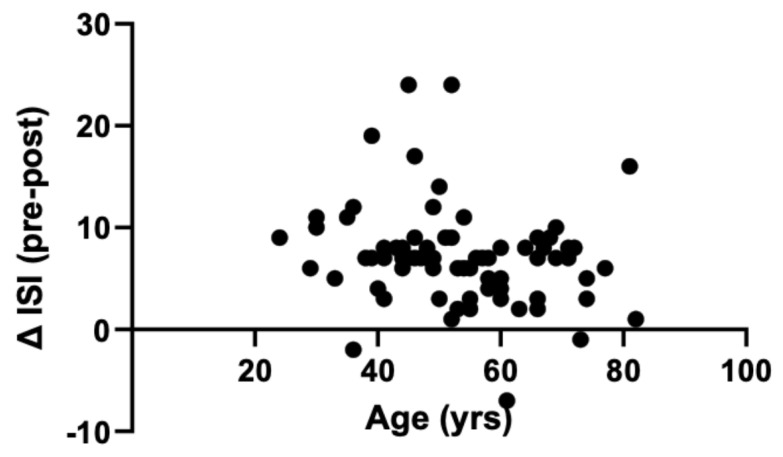
Mean post-30-day change in ISI scores, separated by age. There is a weak but significant negative correlation between patient age and change in ISI score (*n* = 80). *p* < 0.05 as determined by Spearman’s rank coefficient of correlation.

Patients in the analysis cohort ranged in age from 24 to 82, with a mean age of 53.5 years (Figure 4). There is a weak negative correlation between patient age and mean change in ISI (*p* < 0.05). Mean ISI scores for patients ≥ 65 years of age improved from 17.5 pre-treatment to 10.9 post-treatment. Mean change in ISI for the ≥ 65 years group was 6.6, a clinically meaningful and statistically significant improvement (*p* < 0.0001).

#### 3.1.4. Effect of Race/Ethnicity on ISI

**Figure 5:**
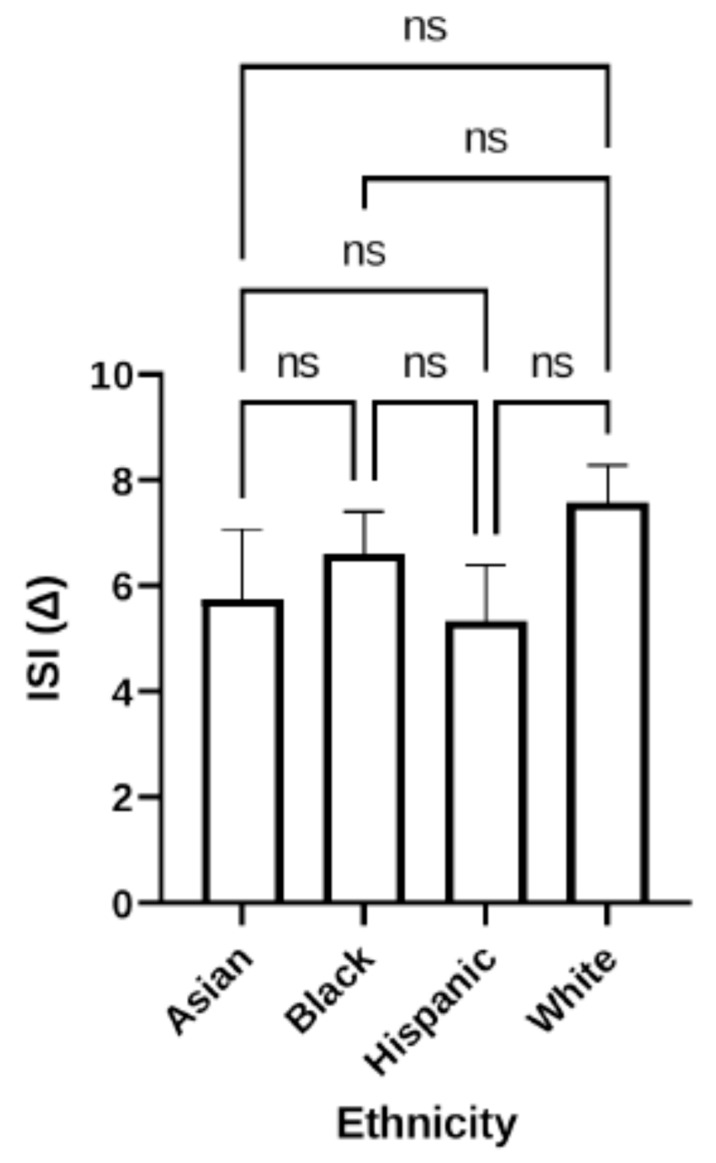
Mean change in ISI score, separated by ethnicity. There is no correlation between ethnicity and mean change in ISI (*n* = 80), as determined by Kruskal–Wallis multiple comparative analysis, with all ethnicities experiencing an improvement in ISI. ns = non-significant.

Potential effects of ethnicity on treatment effectiveness were examined next (Figure 5). Patients fell into four broad categories: Asian, Black, Hispanic, and White. Mean change in ISI over the daridorexant treatment period was analyzed for each of the four categories. ISI improved by 5.8 (Asian), 6.6 (Black), 5.3 (Hispanic), and 7.6 (White), respectively. No significant correlation was determined between mean change in ISI scores and ethnicity.

#### 3.1.5. Impact of Dose on ISI

**Figure 6:**
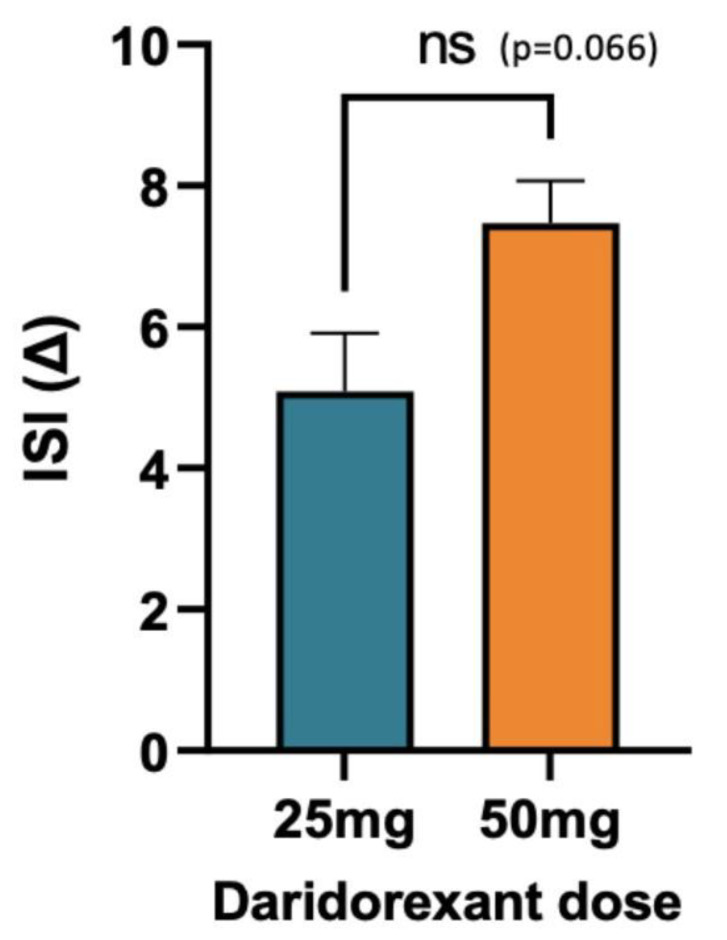
Mean change in ISI score, separated by daridorexant dosage level. There is a non-significant improvement in mean ISI score when taking daridorexant at the 50 mg dose (*n* = 68) versus the 25 mg dose (*n* = 12). Mean changes in ISI scores are 5.1 (25 mg) and 7.5 (50 mg). ns = non-significant with a *p* = 0.066, as determined by Mann–Whitney non-parametric ranked sum test.

Patients treated with 50 mg (*n* = 68) experienced a mean change in ISI of 7.5, while patients treated with 25 mg of daridorexant (*n* = 12) experienced a mean change in ISI of 5.1 (Figure 6). This showed a trend toward improved effectiveness with the higher dose, but given the number of patients in this analysis, it did not meet statistical significance.

#### 3.1.6. Effect of CPAP Therapy

**Figure 7:**
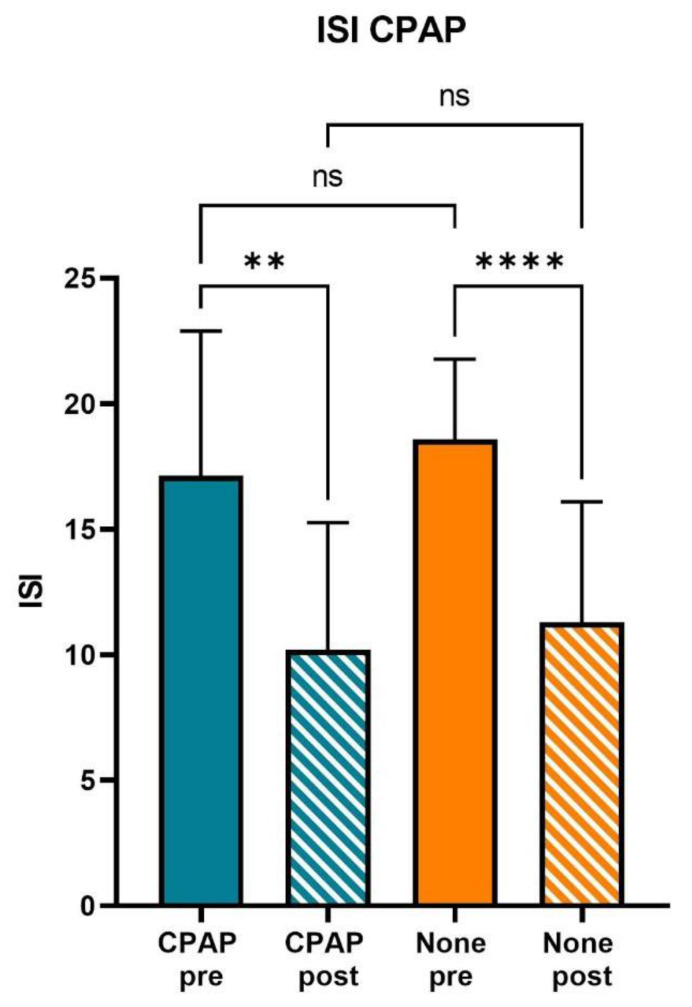
Mean post-treatment change in ISI score for CPAP (*n* = 15) patients versus non-CPAP patients (*n* = 61). ** denotes *p* < 0.01, and **** denotes *p* < 0.0001, as determined by Kruskal–Wallis multiple comparative analysis. ns = non-significant.

A total of 15 of the 80 patients analyzed suffered from obstructive sleep apnea (OSA) and comorbid insomnia (COMISA) and were being treated with CPAP. It is important to understand whether such patients can still expect to receive similar benefits from daridorexant treatment as non-CPAP insomnia patients. Patients treated with dental appliance (*n* = 2) or Inspire implant (*n* = 2) were excluded from the analysis. Analysis showed that the CPAP patients had a mean pre-treatment ISI of 17.1, and a mean post-treatment ISI of 10.2 (Figure 7). This translates to a statistically significant improvement in mean ISI score of 6.9 (*p* < 0.01). Non-CPAP patients (*n* = 61) had a pre-treatment mean ISI of 18.6 and a post-treatment mean ISI of 11.3, resulting in an improvement in mean ISI of 7.3 (*p* < 0.0001). There is no significant difference in responses between CPAP and non-CPAP patients.

#### 3.1.7. Number of Prior Therapies

**Figure 8:**
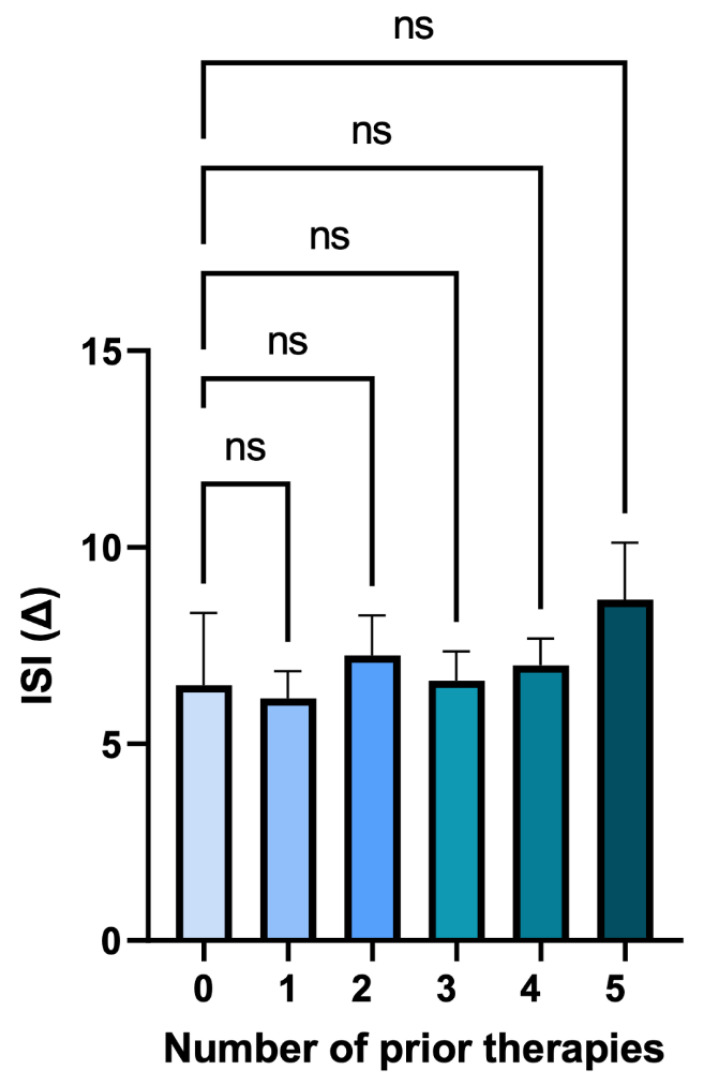
Mean change in ISI score, separated by number of prior insomnia medications. Zero previous drugs (*n* = 6) Δ ISI = 6.5, one previous drug (*n* = 19) Δ ISI = 6.2, two previous drugs (*n* = 20) Δ ISI = 7.25, three previous drugs (*n* = 13) Δ ISI = 6.6, four previous drugs (*n* = 6) Δ ISI = 7.0, five previous drugs (*n* = 3) Δ ISI = 8.7. ns = non-significant. There is no correlation between the number of prior medications taken by each patient for insomnia and the mean change in ISI over the ≥ 30-day daridorexant treatment period. Results based on Kruskal–Wallis multiple comparative analysis.

In total, 11 of the 80 patients in the analysis cohort had no data available for the number of previous medications to treat insomnia and were excluded from this specific analysis. Further, two patients reported six or more previous medications and were excluded from the analysis since the data sets (each *n* = 1) are too small to be considered statistically relevant. The overwhelming majority of patients (*n* = 61) included in this retrospective analysis had been treated with one or more prior medications before starting daridorexant, while six patients were known to have never been previously prescribed any medications for insomnia. The results show no significant correlation between the number of prior therapies and mean change in ISI (Figure 8).

#### 3.1.8. Prior Duration of Insomnia Treatment

**Figure 9:**
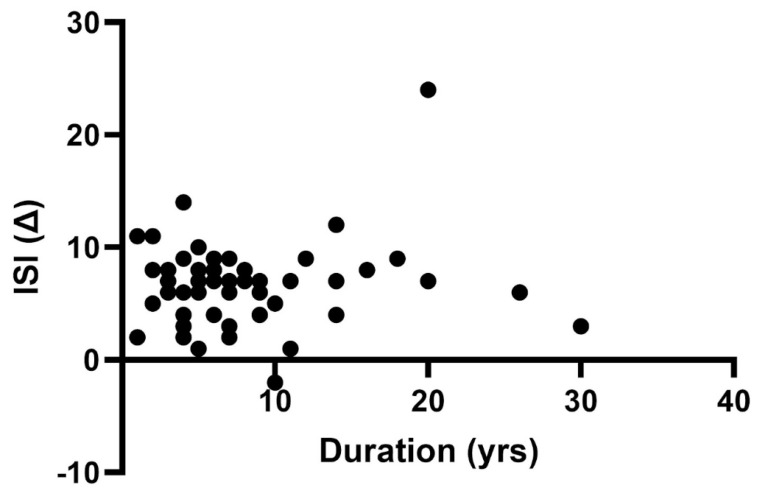
Mean change in ISI score, separated by prior duration of insomnia (*n* = 59). There is no correlation between prior duration of insomnia (in years) and daridorexant benefit as determined by mean post-change in ISI after at least 30 days of treatment. Data represent the degree of confidence as determined by Spearman’s rank coefficient of correlation.

Depending on the medication, the duration of prior insomnia treatments may sometimes lead to rebound insomnia, potentially impacting the evaluation of the new drug’s effectiveness upon switching medications [34,35]. In the current analysis group, 59 out of the 80 patients had prior insomnia duration data and had been previously treated with other hypnotic medications and were thus included (Figure 9). Insomnia duration for this patient cohort ranged from 1 year up to 30 years, with a mean duration of 8.0 years, and a median of 6 years prior to initiation of daridorexant treatment. Despite the wide range, results showed no significant correlation between prior duration of insomnia and mean change in ISI score with daridorexant treatment.

### 3.2. Subjective Measures

#### 3.2.1. Subjective Measures of Daytime Function

Measures of daytime function were self-reported via a sleep diary, both prior to switching and following 30 nights of daridorexant treatment (Table 2). All 80 patients analyzed had self-reported on measures of daytime function prior to being prescribed daridorexant as part of the physicians’ usual clinical practice. The majority of reporting patients experienced one or more of the following symptoms: hypersomnolence (45.0%), daytime fatigue (33.7%), or poor concentration (32.5%) as their primary complaint, while a lower percentage also reported depression (11.3%) and/or anxiety (5.0%). Four of eighty patients reported no adverse effects prior to switching but expressed a desire to switch for another reason (three of these patients stated that they wanted to “get off zolpidem” and the fourth cited “insufficient treatment”, though without effects on daytime function).

Following at least 30 nights of daridorexant treatment, measures of daytime function were obtained for 80 patients (Table 2). A total of 78.7% of patients reported an improvement to some degree in daytime symptoms, with nearly one-third of patients overall (32.5%) reporting that their daytime symptoms were nearly or entirely resolved. Of patients who responded, 12.5% specifically mentioned that they felt increased alertness, and 6.3% reported that their anxiety and/or depression symptoms had improved. 6.3% of patients reported no change in their symptoms.

#### 3.2.2. Subjective Sleep Assessment

**Figure 10:**
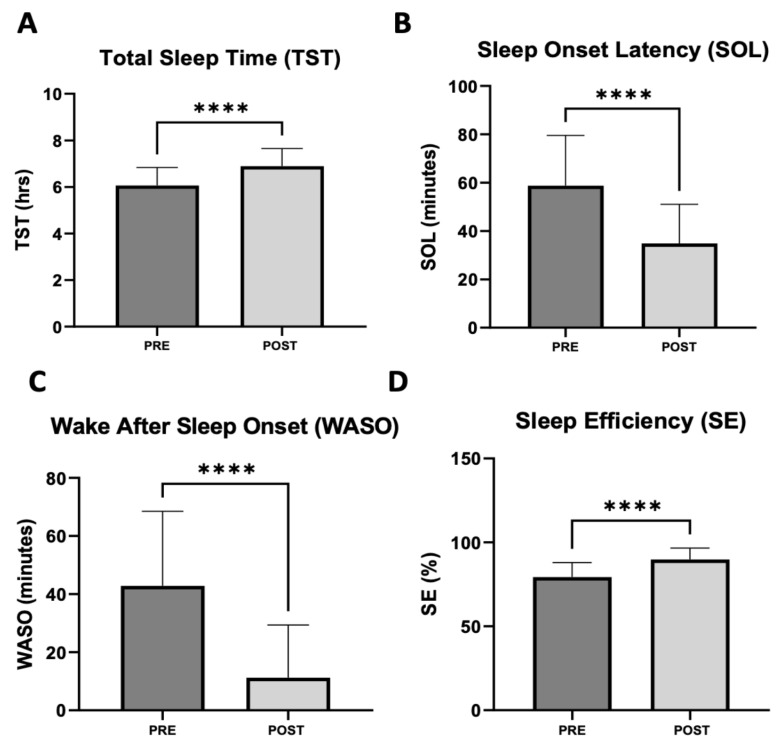
Mean changes in self-reported sleep metrics after treatment with daridorexant (*n* = 44). (**A**) Mean change in TST of 0.9 h (54 min). (**B**) Mean change in SOL of 23.9 min. (**C**) Mean change in WASO of 31.6 min. (**D**) Mean change in SE of 10.5%. All changes in sleep metrics were highly significant and clinically meaningful. Statistical analyses were carried out using Wilcoxon matched pairs signed rank test. **** denotes *p* < 0.0001.

Along with ISI scores, subjective sleep data was collected for 44 patients before and after 30 days or more of daridorexant treatment. The data consisted of four patient-reported parameters: total sleep time (TST), sleep onset latency (SOL), wake after sleep onset (WASO), and sleep efficiency (SE) (Figure 10). There was a statistically significant improvement in all subjective sleep measures. TST demonstrated an increase from a pre-treatment mean of 6 h to a post-treatment mean of 6.9 h, gaining a mean of 54 min of sleep time, with an SEM of 0.06 (*p* < 0.0001). SOL decreased by 23.9 min from a pre-treatment mean of 58.8 min to a post-treatment mean of 34.9 min, with an SEM of 2.4 (*p* < 0.0001). WASO decreased by 31.6 min from a pre-treatment mean of 42.8 min to a post-treatment mean of 11.3 min, with an SEM of 3.2 (*p* < 0.001). SE improved by 10.5% from a pre-treatment mean of 79.3% to a post-treatment mean of 89.8%, with an SEM of 1.1 (*p* < 0.0001).

#### 3.2.3. Sleep Assessment in Patients Treated with BZD GABA-A PAMs versus non-BZD GABA-A PAMs Prior to Switching to Daridorexant

**Figure 11:**
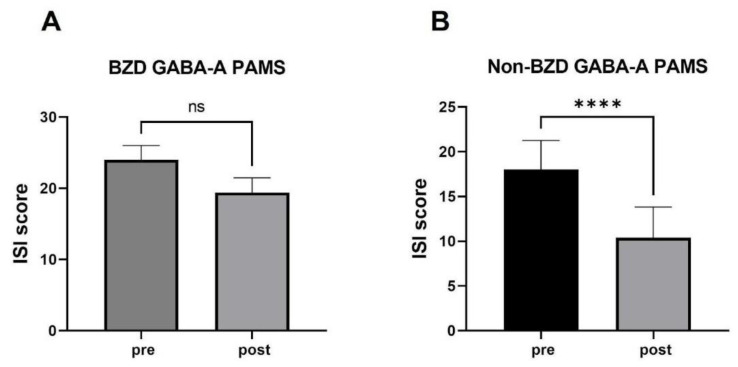
Mean change in ISI before and after 30 days or more of daridorexant treatment with patients who were prescribed (**A**) BZD GABA-A PAMs (*n* = 5) or (**B**) a non-BZD GABA-A PAMs (*n* = 23). **** denotes *p* < 0.0001 as determined by Wilcoxon matched pairs signed rank test. ns = non-significant.

A total of five patients were taking clonazepam prior to initiation of daridorexant treatment. Whilst clonazepam does not have an FDA indication for the treatment of insomnia, it is still sometimes prescribed for patients with comorbid anxiety. These patients reported a mean pre-treatment ISI score of 24.0, and a post-treatment mean ISI score of 19.4, resulting in an improvement in ISI score of 4.6, which was not statistically significant in this small group size, with an SEM of 0.68 (*p* = 0.06). (Figure 11). By contrast, 23 patients taking zolpidem or eszopiclone reported an improvement of 7.6, from a mean pre-treatment ISI score of 18.0 to a post-treatment mean ISI score of 10.4, with an SEM of 0.86 (*p* < 0.0001).

### 3.3. Changes in ISI and Subjective Sleep Measures for Patients Who Discontinued Medication

In the 80-patient cohort with pre- and post-treatment ISI data, 6 patients had discontinued daridorexant treatment after at least 30 days. All 6 were treated with the 25 mg dose of daridorexant for their entire time on the medication and reported an “insufficient response” as to their reason for discontinuation. Mean pre-treatment ISI of 23.0 and post-treatment ISI of 19.7 resulted in a mean improvement in ISI of 3.3 (Table 3). Of those 6 patients, 4 reported sleepiness as a side effect of the treatment, with 3 of those 4 also reporting sleepiness and insufficient response with their previous insomnia medications (clonazepam and doxepin). Subjective sleep measures were recorded for 4 of the 6 patients, and improvements were reported for TST (mean of 36 min), SE (mean of 10.5%), SOL (mean 23 min) and WASO (mean of 20 min).

## 4. Discussion

The ever-expanding array of options for clinicians to consider for the treatment of insomnia is encouraging, and knowledge of the differential patient responses to the various mechanisms of action is essential to maximize therapy. The concept of “turning off wake” via the orexin pathway as opposed to “turning on sleep” via the GABA pathway has been of great interest to the sleep community, and as we move towards a more personalized approach to the treatment of medical conditions, assessing the impact of dose, previously prescribed drug classes, treatment durations, and patient demographics is important.

The American Academy of Sleep Medicine (AASM) Clinical Practice Guidelines recommend only a few of the many available sedating medications for the management of chronic insomnia [2]. Of the BZD GABA-A PAMs, temazepam and triazolam are suggested as a treatment for sleep onset insomnia, and only temazepam is suggested for the treatment of sleep maintenance insomnia. The non-BZD GABA-A PAMs eszopiclone, zaleplon and zolpidem all received a recommendation for the treatment of initiation insomnia, and both eszopiclone and zolpidem received a recommendation for the treatment of maintenance insomnia. Ramelteon, a melatonin receptor agonist, received a recommendation for the treatment of initiation but not maintenance insomnia. Doxepin, a sedating tricyclic antidepressant with antihistaminergic effects, was suggested for maintenance insomnia but not initiation insomnia. Suvorexant was the only DORA approved by the FDA at the time the latest AASM guidelines for chronic insomnia were published, and at that time, it received a recommendation for the treatment of maintenance insomnia. We therefore undertook this analysis to determine the magnitude of the benefit of switching from sleep-inducing pharmacotherapy to wake-antagonizing pharmacotherapy, and to determine whether there were any trends with respect to patient demographics which might inform clinician selection. Most of the patients in our analysis had previously been incomplete responders to a variety of different medications and ultimately had clinically meaningful and significant improvements in ISI scores after treatment with daridorexant for 30 days or more. The cohort overall dropped from moderate insomnia, with a mean ISI of 18 to sub-threshold insomnia with a mean ISI of 11. Longer-term data which was available for 59 of 86 patients demonstrates a consistent and durable response for this medication. It is encouraging that only two patients discontinued the medication between the 1- and 3-month time period.

Interestingly, male patients tended to benefit slightly more than female patients with a mean ISI change of 8.8 compared with 6.2. Whilst there is no working hypothesis for the greater response seen in males, it does raise the question of whether the orexin system is inherently different in male insomnia patients compared with that of female insomnia patients. The small sample size of this group and presence of COMISA limit any firm conclusions, however.

Patients of all ages tended to benefit from switching treatments, but there was a weak decreasing effect of DORA therapy with age, which could suggest that the orexin system might play less of a role in geriatric insomnia patients compared with younger individuals. It is worth noting that the mean age of the cohort was 53.5 years, and only 11 patients under the age of 40 were present so once again the sample size makes it difficult to fully assess this factor. It would be interesting to compare two cohorts of significantly different ages to see if this finding is replicated.

It is also worth noting that there was no differential effect amongst the patients with respect to prior number of medication treatment trials. This is perhaps because many of the sedating medications which do not affect orexin are similar in terms of their mechanism of action, with anti-GABAergic or antihistaminergic pathways predominating. Detailed analysis was not performed with respect to the exact type of medication treatments patients had undergone. Given the sample size, it would likely not have reached statistical significance. This is an important aim for further research, however.

Similarly, the duration of insomnia did not seem to impact the magnitude of the effect size for daridorexant. While data regarding the duration of insomnia treatment versus duration of insomnia symptoms was not compared, it is striking that patients showed a marked response even after many years of suffering from fragmented sleep. This is very curious and worth exploring in more detail.

The reasons for wanting to switch insomnia therapy are informative. Of the 63 patients for whom such data were available, most reported that their original regimen was ineffective. A slightly smaller but substantial number of patients reported side effects from the prior medication or that their prior medication caused next-day somnolence. Some reported complex sleep-related behaviors and a few specified that they “no longer wanted to take zolpidem”. It is worth noting that Behavioral Sleep Medicine (BSM) interventions received by patients prior to inclusion in this study varied widely, and ranged from standard sleep education to internet-delivered principles of CBT-I. In some cases, formal CBT-I or Brief Behavioral Treatment for Insomnia was received. A lack of standardization in this population is reflective of the profound lack of qualified BSM providers nationwide.

There was a non-significant improvement in ISI when comparing the 50 mg dose to the 25 mg dose (*p* < 0.06). In prior data, there was not an increased risk of side effects at the 50 mg dose, so even though the change did not reach statistical significance, it is worth considering starting at the 50 mg dose (and/or increasing to the 50 mg dose if there is incomplete response at the lower dose) [25,26].

Another consideration when selecting treatment for insomnia is the effect of daytime functioning. While earlier treatment focused almost exclusively on sleep fragmentation, in 2014 the American Psychiatric Association recommended the diagnostic criteria for chronic insomnia be updated to specifically include symptoms of daytime function impairment [36]. These recommendations led to a greater emphasis on improving daytime function as newer drug classes were developed [36,37,38,39]. Our data showed an improvement in self-reported daytime functioning when switching to daridorexant, which is logical given the increased specificity of its target.

An additional point to consider in this analysis is that daridorexant treatment has not been studied in patients with severe obstructive sleep apnea, and one of the limitations of this analysis, aside from the small sample size of patients who were on CPAP therapy, is that such patients in this analysis were not stratified according to the severity of sleep apnea.

It is worth discussing the 6 of 80 patients who switched to daridorexant but did not notice a substantial improvement in sleep. Following at least 30 days of treatment, these patients reported “insufficient response”, all at the 25 mg dose. 4 of these 6 patients had been on clonazepam immediately prior to directly switching treatments, and 2 patients had been on doxepin. It was unclear as to the reason why these patients discontinued daridorexant prior to being given the opportunity to increase to the 50 mg dose. It is also possible that the immediate discontinuation of a BZD GABA-A PAM caused a transient rebound in symptoms and may have impacted the overall effect of daridorexant. Abrupt discontinuation of BZDs can lead to both withdrawal and rebound insomnia, reviewed recently by Hintze and Edinger [40]. This is an important point of consideration for clinicians when switching drug classes.

A final confounding factor when interpreting these data is that the majority of the patients were prescribed daridorexant because of a lack of effectiveness of prior medication. There may indeed be an anticipatory effect of a perceived advantage of “the newest medication”. Conversely, there may have been pessimism in some patients who had rounds of multiple drugs in their past and might have felt that “nothing is going to work”.

## 5. Conclusions

These data support the growing awareness that insomnia disorder is heterogeneous and complex. Based on these preliminary results, switching to daridorexant may be a safe and effective treatment alternative for insomnia patients with incomplete or insufficient response to current therapies. Further studies with a longer duration of the observation period including a broader spectrum of outcomes are desirable. Additional research should focus on the effect of DORAs on CPAP adherence, and whether there are any insomnia subtypes that may be more or less responsive to the various pharmacologic classes.

## Figures and Tables

**Table 1:** Sample characteristics of the patient population. Cohort 1 consisted of medical records collated by Dr. Williams and Cohort 2 consisted of medical records collated by Dr. Cue. For both cohorts, the study collection period was from 8 June 2022 until 9 November 2022. After exclusion criteria were applied (see Figure 1), a total of 80 patients constituted the final sample population for this study.

Descriptor	Patient	Patient	Total Sample
	Cohort 1	Cohort 2	Population
**Number of Subjects Analyzed**	59	21	80
Male	20	8	28
Female	39	13	52
**Age range (years)**	24–82	30–74	24–82
Mean age (SD)	53.1 (12.6)	53.9 (14.3)	53.5 (13.0)
Age 64 and under	46	15	61 (76.3%)
Age 65 and older	13	6	19 (23.8%)
**Race/Ethnicity**			
Asian	4	0	4
Black	8	2	10
Hispanic	8	1	9
White	39	18	57
**Insomnia Severity Index (ISI) score**			
Mean pre-daridorexant	18.1	18.0	18.0
Range (SD)	9–26 (2.8)	5–28 (5.3)	5–28 (4.1)
Mean post-30-night daridorexant	11.3	10.7	11.0
Range (SD)	4–22 (3.8)	0–20 (6.4)	0–22 (4.9)
Mean within-patient change in ISI	−6.8	−7.3	−7.0
Range (SD)	1–14 (2.7)	−7–24 (8.1)	−7–24 (4.7)

(SD) is standard deviation.

**Table 2:** Subjective Measures of Daytime Function.

Number of Subjects Reporting	% Total Subjects (of 80)	Subjective Measures of Daytime Function Pre-Treatment	Number of Subjects Reporting	% Total Subjects (of 80)	Subjective Measures of Daytime Function Post-Treatment
36	45.0	hypersomnolence/severe to moderate sleepiness	37	46.2	fatigue/sleepiness improved
27	33.7	daytime fatigue	26	32.5	fatigue/sleepiness resolved/nearly resolved
26	32.5	poor concentration	10	12.5	increased alertness
9	11.3	depression	5	6.3	anxiety/depression improved
4	5.0	anxiety	5	6.3	no change in symptoms
4	5.0	none	4	5.0	no change/no symptoms

**Table 3:** Pre- and post-treatment ISI and subjective measures of sleep (TST, SOL, WASO and SE) in the six patients that discontinued daridorexant after 30 days of treatment. - denotes no available data in the medical records for the patient.

	Insomnia Severity Index			Subjective Measures of Sleep			
Patient	Pre- Treatment ISI	Post- Treatment ISI	Improvement in ISI	Improvement in TST (min)	Improvement in SOL (min)	Improvement in WASO (min)	Improvement in SE (%)
1	26	21	5	24	30	20	12
2	22	19	3	30	30	0	11
3	21	19	2	60	30	30	12
4	26	22	4	30	0	30	7
5	21	19	2	-	-	-	-
6	22	18	4	-	-	-	-
MEAN	23.0	19.7	3.3	36	23	20	10.5

## Data Availability

The data presented in this study are available on request from the corresponding author.
